# An Electrochemical Dopamine Assay with Cobalt Oxide Palatinose Carbon Dots

**DOI:** 10.3390/molecules30020413

**Published:** 2025-01-19

**Authors:** Ram Chandra Nepal, Elif S. Seven, Roger M. Leblanc, Charles C. Chusuei

**Affiliations:** 1 Department of Chemistry, Middle Tennessee State University, 440 Friendship Street, Murfreesboro, TN 37132, USA; rcn2x@mtmail.mtsu.edu; 2 Department of Chemistry, University of Miami, 1301 Memorial Drive, Coral Gables, FL 33146, USA; ess133@miami.edu (E.S.S.); rml@miami.edu (R.M.L.)

**Keywords:** carbon dots, cyclic voltammetry, Raman, graphene defects, dopamine

## Abstract

Elevated dopamine (DA) levels in urine denote neuroblastoma, a pediatric cancer. Saccharide-derived carbon dots (CDs) were applied to assay DA detection in simulated urine (SU) while delineating the effects of graphene defect density on electrocatalytic activity. CDs were hydrothermally synthesized to vary graphene defect densities using sucrose, raffinose, and palatinose, depositing them onto glassy carbon electrodes (GCEs). Co_3_O_4_ nanoparticles (NPs) were encapsulated by the CDs. Cyclic (CV) and linear sweep (LSV) voltammetry measurements were obtained, drop-casting the CDs onto GCEs and measuring DA in a phosphate-buffer solution (pH = 7). DA had an oxidation peak at +0.2 V with SucCDs, with the highest current correlating with the highest defect density. PalCD-Co_3_O_4_ exhibited the largest signal for DA detection in simulated urine (SU) using the oxidation peak at +0.5 V; the composite had a lower defect density compared to SucCD-Co_3_O_4_. The Co_3_O_4_-PalCDs had a DA detection range of 1 to 90 µM with an LOD of 0.88 μM in SU. SEM-EDX analysis of the electrode surface revealed semi-spherical structures with an average particle diameter of 80 ± 19 nm (n = 347) with PalCDs decorating the Co_3_O_4_ NPs. XRD characterization showed the incorporation of PalCD and Co_3_O_4_ within the composite. XPS showed electron density donation from the PalCD to Co_3_O_4_.

## 1. Introduction

Dopamine (DA) is a well-known neurotransmitter which has a significant role in coordinating the essential activities of the entire human system, such as learning behavior, emotion, and movements. The imbalance in the concentration of this biomolecule has been implicated in chronic diseases like Parkinson’s disease, dementia, epilepsy, schizophrenia, and neuroblastoma. Neuroblastoma, a pediatric tumor originating from sympathetic nervous crest cells, is marked by the elevated urinary excretion of DA [1]. Multiple analytical approaches, such as colorimetry [2], HPLC coupled with fluorescence detection [3], micellar liquid chromatography [4], and liquid chromatography–tandem mass spectrometry and spectrophotometry [5], have been used to detect the DA levels in body fluids. While certain techniques can precisely measure dopamine concentrations, they are often expensive and complex. In contrast, electroanalytical methods are cost-effective and require simple setups. Developing a sensor that is both sensitive and selective to common interfering compounds is essential. Khamlichi et al. were the first to prepare a sensor capable of detecting DA within the practical 10–120 µM range in a phosphate-buffer solution (PBS at pH 7) using a leucine-modified sol–gel carbon electrode [1]. More robust composite materials have been explored involving non-perishable components to assay DA levels [6]. The assays of these past studies were limited to quantifying DA in PBS. Carbon nanomaterials such as carbon dots, carbon nanotubes, and graphene have garnered significant interest in recent decades for their technological utility in electrocatalysis for biosensing and fuel cell applications in water-based solutions [7]. However, their often undefined shape and structure pose challenges in understanding how their electrocatalytic activity is influenced by factors such as surface defects, exposed graphene structures, and electroactive area. Despite these challenges, nanostructured carbon remains a popular choice as an electrocatalyst due to its affordability, large specific surface area, and high carrier mobility [8]. The exceptional mobility of electrons in graphene is attributed to its sp^2^ hybridization, which provides an additional electron to the π bond. These π electrons, being delocalized even at room temperature, contribute to the material’s high conductivity [9].

Defects play a role in the electrochemical sensitivity of analytes, but the literature is replete with conflicting examples of increased graphene defect density within the electrocatalyst materials, measured by the relative sp^3^-to-sp^2^ hybridized carbon ratio (typically quantified by the intensities of the Raman D-to-G integrated peak area band ratios) accompanying either increased or decreased redox activity; a recent carbon nanomaterial literature review has resolved this conundrum by pointing out that the correlation of graphene defect density within the carbon nanomaterial is only relevant within the Kislenko range where redox activity is close to the electrochemical Fermi level, between −0.2 V and +0.3 V [10,11]. We postulate that this same trend will hold in this current study.

Another challenge of correlating structure–property relationships of carbon nanomaterials (nanotubes and 2D graphene) is the interference of change in graphene curvature in the accurate experimental measurement of sp^3^ structures. Pristine graphene is typically composed of sp² hybridized carbon atoms arranged in a hexagonal lattice. In its Raman spectrum, two prominent peaks are observed: the D band (sp^3^ carbon) appears at ~1300 cm⁻^1^ and the G band (sp^2^ carbon) at ~1600 cm⁻^1^. The areas under these peaks provide insights into their respective concentrations. Although the Raman spectra are collected from a microscopic portion of the sample, the results can often be extrapolated to represent the entire bulk material. Gupta et al. [12] reported that curvature on a graphene sheet can induce a small D band in which carbon nanotubes, when exceeding a critical diameter, undergo spontaneous collapse into flattened structures. Picheau et al. showed that flattened multi-walled carbon nanotubes exhibited a false D band intensity increase in the Raman spectrum even though the sp^3^ defect density remained unchanged [13], suggesting that past studies in which graphene changes in curvature may have occurred will need to be re-examined for accurate defect quantification. In this study, the Raman spectral analysis of both flattened and cylindrical carbon nanotubes revealed a distinctive and intense D band, even when there was no apparent lattice disorder. The D band was induced by changes in curvature near edge cavities caused by the collapse of the nanotube structure, even though the overall framework remained intact. Of the three types of carbon nanomaterials (0D carbon dots, 1D carbon nanotubes, and 2D graphene), carbon dots are the least susceptible to curvature change given their spherical morphology. For this reason, we have isolated our composite synthesis to involve only CDs, which avoid graphene curvature changes and ensure accurate graphene defect density measurements via Raman spectroscopy. This approach was successful in showing the reason why graphene defect density did not correlate with saccharide CD sensitivity for detecting acetaminophen since the oxidation peak voltage used to do so (+0.549 V) resided outside the Kislenko range.

Tethering of Co_3_O_4_ nanoparticles (NPs) to single-walled carbon nanotubes has been shown to increase the electrocatalytic activity for the detection of DA in bovine serum samples [14]. This present study seeks to develop an optimized CD-Co_3_O_4_ composite for DA analysis in simulated urine (SU) for practical neuroblastoma screening. We utilized carbon dots synthesized from sucrose (SucCD), palatinose (PalCD), and raffinose (RafCD) and applied them onto working glassy carbon electrode surfaces (GCEs) for DA cyclic voltammetry (CV) and linear sweep voltammetry (LSV) measurements. After the initial evaluations of these CDs in PBS, composites of these CDs with Co_3_O_4_ NPs were prepared for DA measurements in SU. In making the optimized composite, the effective minimum dose of Co_3_O_4_ was incorporated to prevent extraneous defects from being introduced to the CD structure, thereby hampering the structure–property study. For this reason, a 1:1 Co_3_O_4_:CD preparation was used for the composite syntheses with the array of CDs (SucCD, PalCD, and RafCD); this ratio was optimal for the CoO-multi-walled carbon nanotube composite in a previous study for assaying DA [15]. X-ray photoelectron spectroscopy (XPS)/EDX data showed no Co_3_O_4_ at the topmost surface of the CD-CD working electrode surface in the final, optimized sensing composite produced (vide infra). Our previous preliminary studies showed that altering the type of saccharide precursor for CD synthesis was sufficient to systematically control the graphene defect density readily measurable with Raman spectroscopy in order to examine this variable’s effect on electrochemical sensitivity for acetaminophen detection [16].

## 2. Results and Discussion

**Figure 1:**
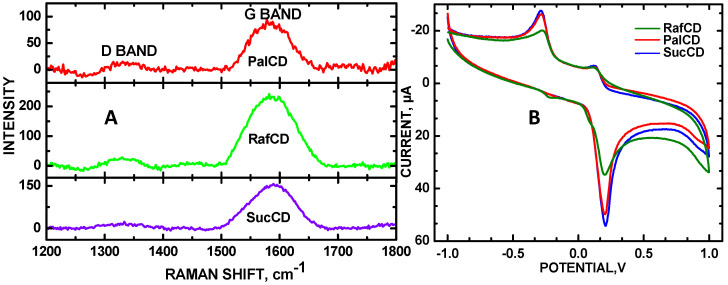
(**A**) Stackplot of Raman spectra in the D and G band region of PalCDs, RafCDs, and SucCDs; (**B**) CVs of 0.4 mM DA with SucCDs, PalCDs, and RafCDs deposited on the GCE in PBS, pH = 7, 50 mV/s scan rate.

**Figure 2:**
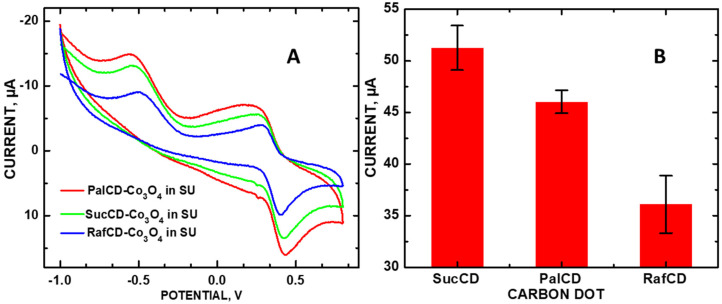
(**A**) CVs of CD-Co_3_O_4_ composites deposited onto GCEs measuring 40 µM DA in SU, 50 mV/s; and (**B**) bar graphs of their measured currents with error bars.

Graphene defects of the CDs were quantified from the peak area of the D band, and pristine graphene was quantified from the G band, respectively [17]. The D band represented sp^3^ hybridization, indicative of disorder within the graphene sheet of the carbon nanomaterial, whereas the G band signified sp^2^ hybridization, reflecting the orderly arrangement of carbon atoms in graphene. The ratio of the D band to the G band differed among the CDs (with I_D_/I_G_ ratios in parentheses) in descending order: SucCD (0.185) > PalCD (0.117) > RafCD (0.0976) (Figure 1A). Integrated peak areas of the Raman D and G bands are shown in Figure S1 in the Supplemental Information Section. SucCD exhibited the highest defect density, correlating with maximum electrochemical sensitivity for measuring 0.4 mM DA in PBS. In theory, defects within the sp^3^ carbon serve as an electron sink, enhancing electron transfer. Specifically, the anodic peak current, measured in a 0.3 mM DA solution in PBS at pH 7, showed a descending current trend: SucCD (51 µA) > PalCD (46 µA) > RafCD (36 µA) (Figure 1B), where the magnitude of anodic currents increased (Figure 2A) with higher defect density (Figure 1A). This observation aligns with predictions from density functional theory (DFT) calculations using the Gerischer model, which suggests that for aqueous solutions, an increase in sp^3^ carbon defects enhances electron transfer near the Fermi level [10,11].

**Figure 3:**
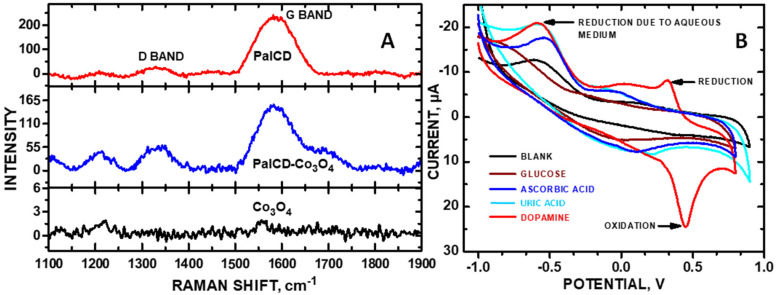
(**A**) Raman spectra of PalCD-Co_3_O_4_, SucCD-Co_3_O_4_, and RafCD-Co_3_O_4_; and (**B**) CV selectivity measurements of PalCD-Co_3_O_4_ toward 0.4 mM DA, glucose, ascorbic acid, and uric acid in SU are shown.

The incorporation of Co_3_O_4_ NPs with each of the saccharide CDs led to an even greater increase in CV and LSV DA signals. This modification resulted in the redox potential to shift from +0.2 to +0.5 V when transitioning from the phosphate-buffer solution (PBS) to SU due to the latter solution’s increase in resistance relative to PBS as a result of additional solutes (Figure 2B). The reduction peak in Figure 2B at −0.5 V is due to the PBS, verified by a blank experiment. The departure from the correlation of anodic current intensity with defect density is consistent with observations made for other carbon nanomaterials, noting that this effect is pronounced only with redox reactions close to the Fermi level [11]. Figure 3A shows Raman spectra of the PalCD-Co_3_O_4_ components; clearly, the addition of the Co_3_O_4_ to the PalCDs resulted in a defect density increase, which was greater than that of pristine PalCDs as indicated by the pronounced increase in peak intensity at 1350 cm^−1^ for PalCD-Co_3_O_4_ (Figure 3A). In this instance, an increase in defect density is observed accompanying the increase in the redox signal at +0.470 V. Given the fact that the anodic signal is greater than +0.3 V, the increased current is attributable to the Co_3_O_4_ NPs enhancing redox activity.

The pronounced Raman peak observed at 1213 cm⁻^1^ is an artifact of the Co_3_O_4_ NPs (Figure 3A). The Raman D band appeared at 1350 cm^−1^ (denoting defects), and the G band at 1585 cm^−1^. The bare Co_3_O_4_ also made a small contribution to the G band within the PalCD-Co_3_O_4_ composite, the integrated peak area of which was subtracted out to make reliable comparisons of the graphene defect density. Noteworthy is the fact that the peak area of the D band increased in the PalCD-Co_3_O_4_ as compared to pristine PalCD (Figure 3A), which may contribute to the increased electrochemical sensitivity for reactions within the Kislenko voltage range. The Raman spectra of PalCD to PalCDs-Co_3_O_4_ showed that graphene defect density increased from I_D_/I_G_ = 0.117 to 0.227. PalCD-Co_3_O_4_ deposited onto the glassy carbon electrode (GCE) was also selective toward DA and against glucose, ascorbic acid, and uric acid, respectively; 0.4 mM concentrations of each analyte were assayed in these CVs (Figure 3B). DA showed a distinct oxidation peak, while ascorbic acid and uric acid showed insignificant peaks at different potential regions. Among the interfering molecules, DA is the only analyte that displays a reduction peak (+0.319 V), further highlighting the composite’s selectivity as an electrocatalyst.

**Figure 4:**
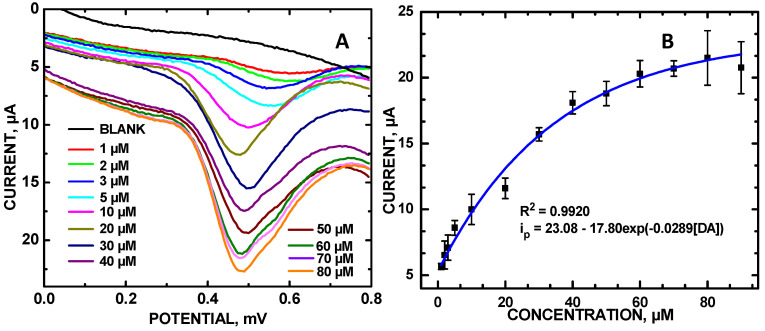
(**A**) LSVs of DA in SU; and (**B**) calibration plot of DA in SU using PalCD-Co_3_O_4_/GCE as the working electrode.

Raw LSV data for the oxidation of DA in SU (Figure 4A) were used to generate the exponential calibration curve of DA with PalCD-Co_3_O_4_ (Figure 4B). However, the SucCD-Co_3_O_4_ did not have the highest redox signal within the saccharide series even though the composite still had the highest defect density (I_D_/I_G_ = 0.420; Figure S4A in the Supplemental Information). The redox potential shifted to a higher voltage, from +0.2 to +0.5 V, in the changing of the solution media from PBS to SU, owing to the greater number of solutes present in SU as compared to PBS, resulting in higher electrical solution resistance. The CD-Co_3_O_4_ composites for 0.4 mM DA detection exhibited the descending anodic current at +0.450 V in the following order: PalCD-Co_3_O_4_ > SucCD-Co_3_O_4_ > RafCD-Co_3_O_4_. Defect density calculations reveal an overall increase in defects, with the PalCD-Co_3_O_4_ composite having a lower defect density (I_D_/I_G_ = 0.227) compared to the SucCD composite (I_D_/I_G_ = 0.420). However, the lowered defect density did not correlate with its improved signal detection for DA. Since the redox potential was outside the −0.2 to +0.3 V range, reported by Kislenko et al. [10], the case of PalCD-Co_3_O_4_ having a reduced defect density (as compared to that of SucCD-Co_3_O_4_) is consistent with the predicted theory. A similar phenomenon (deviation from increased graphene defect density did not lead to higher current) was observed by Chusuei et al. [16] in the monitoring of acetaminophen with various graphene-containing electrocatalysts, which had a redox detection of +0.549 V.

Raw LSV signals for the oxidation of DA in SU (Figure 4A) was used to generate the calibration curve, plotting concentration versus current. Analytically, a calibration curve is found to fit an asymptotic exponential fit where the current for the planar electrode is as follows: i_p_ (μA) = 23.08–17.80∙exp (−0.0289[DA]) with R^2^ = 0.9920 effective for the 1–90 µM DA concentration range (Figure 4B). The limit of detection was 0.88 μM. From a Randles–Sevçik analysis, a plot of i_p_ versus v^1/2^ (square root of the scan rate) yielded a linear plot (R^2^ = 0.9974), indicating a diffusion-controlled electrocatalytic reaction between DA and the PalCD-Co_3_O_4_/GCE surface (Figure S3A). Considering the observed reversible reaction with i_p_ = 2.69 × 10^5^ A⋅D^1/2^⋅n^3/2^⋅v^1/2^⋅C_0_ [18], using the area of the GCE (A = 0.1962 cm^2^), n = 2 electrons for the formation of oxidized dopamine orthoquinone, C_0_ = 0.1 mM, and slope = 3.79 × 10^−4^ A (V∙s)^−1/2^, a diffusion coefficient of αD = 7.41 × 10^−5^ cm^2^∙s^−1^ was calculated for DA oxidation. We attribute the deviation (plateauing) from linear behavior from the saturation of redox sites for the DA analyte on the PalCD-Co_3_O_4_/GCE surface.

Considering a 24-h urination volume of 200 mL and a 400 µg amount of dopamine within that period in the total urine sample, typical of a healthy 4-year-old child [19], the molarity of dopamine in normal urine is [4.00 × 10^−4^ g ÷ 189.64 g/mol ÷ 0.200 L =] 10.55 µM, which the electrode can easily detect. The elevated dopamine level, due to neuroblastoma, could be significantly higher than that value. Hence, the PalCD-Co_3_O_4_ composite is suitable for monitoring DA within a practical range. In contrast, while SucCD-Co_3_O_4_ had a wider analytical detection range of DA in SU, its limit of detection was higher (10–110 µM) than that of PalCD-Co_3_O_4_ (1–90 µM); no signal was detectable under 10 µM DA. The SucCD-Co_3_O_4_ followed an exponential fit for detecting DA in SU: i_p_ = 75.53–77.65∙exp(−0.0124[DA]), R^2^ = 0.9997.

**Figure 5:**
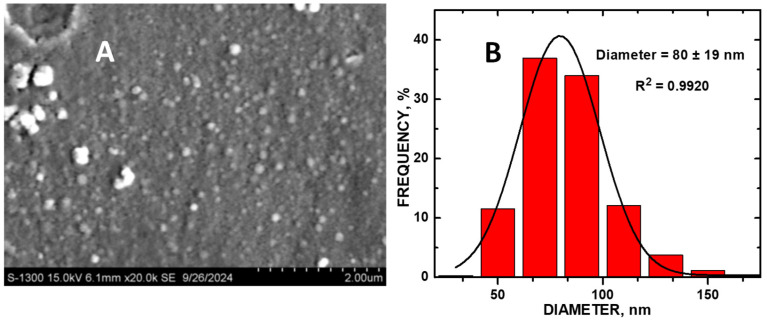
(**A**) Scanning electron micrograph of PalCD-Co_3_O_4_/GCE; and (**B**) size histogram distribution of PalCD-Co_3_O_4_ NPs.

The particulates adsorbed onto the GCE had a semi-spherical shape (Figure 5A,B). The interaction of PalCDs and Co_3_O_4_ was verified with energy dispersive X-ray spectroscopy (EDX) results. An insignificant amount of cobalt was detected in the EDX result, indicating the multilayer formation of CDs around cobalt oxide. Scanning electron microscopy (SEM)-EDX analysis revealed that an average composite particle size was calculated as 80 nm ± 19 (n = 347) with an 18.7 atom % Co present (Figure S2 in the Supplementary Materials). This lowered value as compared to the predicted 75 atom % Co suggests that the PalCDs decorated the Co_3_O_4_ NPs, attenuating an underlying Co K signal measured by the EDX.

**Figure 6:**
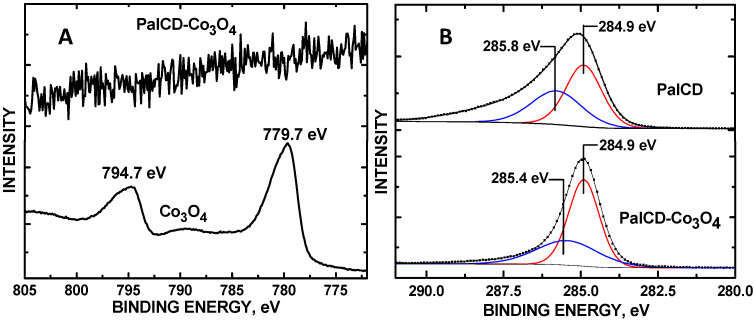
(**A**) Co 2p core levels of PalCD-Co_3_O_4_; and (**B**) C 1s core levels of PalCD and PalCD-Co_3_O_4_.

**Figure 7:**
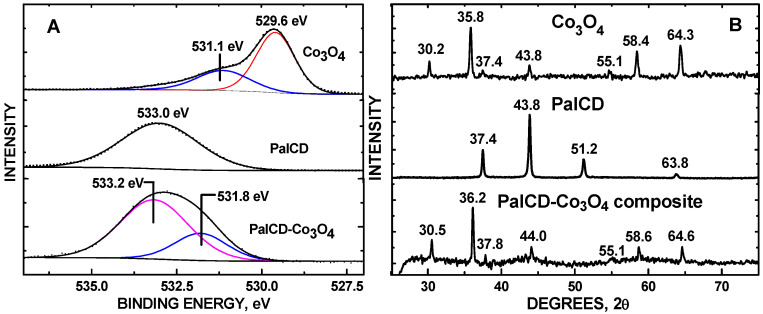
(**A**) O 1s core levels of Co_3_O_4_, PalCD, and PalCD-Co_3_O_4_; and (**B**) XRD of Co_3_O_4_, PalCD, and PalCD-Co_3_O_4_.

The XPS of the Co 2p core level showed no signal for the PalCD-Co_3_O_4_ composite (Figure 6A), signifying that the PalCDs encapsulated the Co_3_O_4_ nanoparticles with a layer 100Å thick within the PalCD-Co_3_O_4_ composite (top); the bottom spectrum of Figure 6A shows core levels for the Co_3_O_4_ NPs used to construct the composite where the Co 2p_3/2_ and 2p_1/2_ binding energies at 779.7 and 794.7 eV, respectively, denote the mixed Co(II/III) oxidation states typical of the material [20]. This lack of signal ensures that electrochemical activity for DA detection emanates predominantly from the CD surfaces in our evaluation of the role of defects. Figure 6B shows C 1s core levels for PalCD and PalCD-Co_3_O_4_. The BE at 284.9 eV matches the alkenyl carbon observed in our previous reports of saccharide CDs. Within the PalCD sample, the C 1s levels at 285.8 eV binding energy (BE) denote a polymeric -(CH_2_CH_2_)_n_- structure within the CDs [21]. As the CDs encapsulated the Co_3_O_4_ NPs, the lowered BE to 285.4 eV indicated increased electron density, likely emanating from the adsorbed O atoms donating electron density to facilitate the bonding of the CDs to it and subsequent encapsulation. Bonded or coordinated O (more electronegative than C), on the other hand, from Co_3_O_4_ would withdraw electron density from PalCD. In support of this mechanism, the O 1s core level of the PalCD-Co_3_O_4_ shows a higher BE, denoting electron deficient density at 531.8 eV, as compared to the corresponding O 1s core level for the Co_3_O_4_ at BE = 531.1 eV (Figure 7A), which we assign to chemisorbed O atoms within the Co_3_O_4_ lattice. The O 1s BE = 529.6 eV matches the literature values for the Co_3_O_4_ metal oxide oxidation state [22,23]. The O 1s BE core level at 533.0 eV is unique to PalCDs; attempts to deconvolute this envelope into additional oxidation states using CasaXPS software algorithms were inconclusive due to the lack of asymmetry in the line shape. The corresponding increase in O 1s BE for PalCD-Co_3_O_4_ (533.2 eV), as compared to PalCD (533.0 eV), is also a result of the electronegative O (from Co_3_O_4_) withdrawing electron density from the PalCD, resulting in bonding. X-ray diffraction (XRD) patterns of the PalCDs showed a crystalline structure with 2θ peaks at 37.4°, 43.8°, 51.2°, and 63.8° (Figure 7B), resembling a dumortierite structure with a pseudohexagonal structure [24]. The XRD peaks of Co_3_O_4_ matched the literature values for this material [25]. XRD showed PalCD and PalCD-Co_3_O_4_ to have an amorphous structure below 2θ = 30°. This is only the second report of such a high level of crystallinity in the literature, with the first emanating from yellow CDs [26]. While there is no coordinate metal in the PalCD, covalent organic frameworks (COFs) [27] are known to display an analogous crystallinity. The Co_3_O_4_ nanopowder showed the expected characteristic peaks for the material. The PalCD-CO_3_O_4_ composite had elements of both PalCD and Co_3_O_4_ with the exception of the missing 2θ = 51.2° peak emanating from PalCD; we postulate that this structural feature associated with this XRD peak within the PalCD was involved in bonding/coordinating with Co_3_O_4_.

## 3. Conclusions

In summary, in the CVs of DA in PBS, the anodic peak current appeared at +0.2 V. SucCDs had the highest defect density and performed best for DA detection. However, SU CD-Co_3_O_4_ composites showed superior performance, with Co_3_O_4_-PalCDs yielding the highest peak current at +0.5 V. After incorporating Co_3_O_4_, the defect density of SucCDs increased from I_D_/I_G_ = 0.195 to 0.420, while in PalCDs, it rose from 0.117 to 0.224. Although a marked increase in defects was observed for SucCD-Co_3_O_4_, PalCD-Co_3_O_4_ still had greater sensitivity owing to the oxidation peak (+0.5 V) lying outside the Kislenko (−0.2 to +0.3 V) range. The PalCD-Co_3_O_4_/GCE had a detection range of 1–90 μM in SU, well within limits for practical neuroblastoma monitoring. Noteworthy is the change in the influence of graphene defects on sensitivity when formulating electrocatalysts, which depended on the location of redox voltage for analyte detection. This general principle may be universal for optimizing graphene-containing electrochemical sensors applicable to a host of analytes. Also noteworthy is the discovery of PalCD’s high crystallinity (CDs are typically amorphous) and its incorporation along with that of Co_3_O_4_ within the PalCD-Co_3_O_4_ structure. The composite had elements of both Co_3_O_4_ and PalCD except for the missing phase at 2θ = 51.2°, which was likely involved in tethering the PalCD to Co_3_O_4_. While EDX revealed the presence of Co in the composite, no signal whatsoever (Figure 6A) was observed in the XPS, indicating that the PalCDs completely encapsulated the Co_3_O_4_ with at least a 100 Å thickness. The lack of XPS signal for Co, while still detectable by EDX, signified that the effective minimum dose of Co_3_O_4_ for DA signal enhancement was achieved, which assured that its presence did not hamper graphene defect density analysis within the CDs. The XPS analysis of the O 1s and C 1s core level shifts in pristine PalCD and Co_3_O_4_ before and after their combination to form the composite revealed that electron density donation from the PalCD to the Co_3_O_4_ occurred, contributing to the bonding between these components.

## 4. Materials and Methods

All reagents were of analytical grade (99% or greater purity). Cobalt oxide (Co_3_O_4_) particles were purchased from Nanostructured and Amorphous Materials (Los Alamos, NM, USA) and used as received. L-dopamine, L-ascorbic acid, phosphate buffer solution (PBS) at pH 7, low-molecular-weight chitosan, sodium bicarbonate, raffinose pentahydrate, palatinose hydrate and sucrose, and uric acid were purchased from Sigma-Aldrich (99% or better purity; St. Louis, MO, USA). HCl and NaOH were purchased from Fisher Scientific (Pittsburgh, PA, USA). Absolute anhydrous ethyl alcohol (AAEA) was purchased from Pharmco-AAPER (Brookfield, CT, USA). Micropolish^TM^ Al_2_O_3_ slurries 0.05 and 1.0 µm in diameter were purchased from Buehler, Ltd. (Lake Bluff, IL, USA). Simulated Normal Urine was obtained from Carolina Biological Supply Company (Burlington, NC, USA).

Three saccharide carbon dots with various surface defect densities were synthesized using a bottom-up approach with a hydrothermal method. They were produced by subjecting 20 mL of 0.3 mM saccharide solutions of raffinose, sucrose, and palatinose, respectively, to hydrothermal treatments. The solution was placed in a Teflon-coated reactor, heated to 200 °C for 30 min, and maintained at that temperature for 5 h. After cooling, the solid material was separated through centrifugation, and the supernatant was filtered to remove fine particles. The pH was adjusted to eight using a saturated NaOH solution. The solution underwent dialysis in 18 MΩ deionized water for 3 days using a one kDa molecular weight cut-off (MWCO) dialysis membrane, with water changes every 10–12 h. Finally, the resulting solution was lyophilized to obtain a solid product [16,28]. The suspension containing CDs and Co_3_O_4_ NPs in a 1:1 mass ratio, with a concentration of 1 g/mL in absolute anhydrous ethanol, was sonicated for 30 min using a 60 W Sharpertek XP Pro SH80-2L sonicator (Pontiac, MI, USA). Subsequently, 10 µL of this suspension was applied to the surface of a glassy carbon electrode. A 2 wt% chitosan in 18 MΩ water was then used as a capping agent to encapsulate the electrode material on the electrode’s surface and allow it to set at 5 °C for 24 h prior to electrochemical analysis.

Electrochemical experiments were carried out using a WaveNano^TM^ potentiostat (Pine Instrument Co., Raleigh, NC, USA) for CV and LSV measurements. The electrochemical experiments were conducted in a three-electrode cell configuration. This setup included a platinum wire as the counter electrode, a Ag/AgCl (3.5 M KCl) electrode as the reference, and a working GCE.

A Horiba Jvon LabRam HR Evolution Raman spectrometer (Irvine, CA, USA) was used to acquire sp^3^- and sp^2^-hybridized carbon spectra to obtain their respective integrated D band and G band peak areas. The Raman spectrometer was employed to quantify the defect density in carbon dots. Each of the carbon dots—SucCDs, RafCDs, and PalCDs—were placed directly onto separate microscopic glass slides for the Raman experiments, requiring no additional sample preparation. These slides, each containing their respective carbon dots, were inserted into the instrument’s case after calibrating it with 534 nm and 785 nm lasers. Initially, the sample was focused, and its position was secured. A 50× magnification lens was chosen to focus the laser onto the sample surface precisely. Settings were adjusted to utilize a 534 nm laser at 5% power, with an acquisition time of 10 s with 12 accumulations. Subsequent spectra were recorded for all the mentioned types of carbon dots. Raman integrated peak areas were determined using OriginPro 8.5 (OriginLab Corp., Northampton, MA, USA).

SEM-EDX was carried out using a Hitachi S-3400 Scanning Electron Microscope equipped with an Oxford Aztec INCA X-Act spectrometer (Westford, MA, USA) operated at 15 kV and 20,000× magnification. Copper tape and sputtering the electrode with Au and Pd were employed to reduce the charging effects that hamper the acquisition of high-resolution imaging due to surface charging from GCE housing. ImageJ software (ver 1.46r, Java 1.6.0; National Institutes of Health, Bethesda, MD, USA) was used to obtain average diam and histogram calculated data with Origin Pro 8.5.

XPS was acquired using a Thermo Scientific Nexsa G2 spectrometer (Waltham, MA, USA) equipped with a hemispherical analyzer and a 128-channel detector, an Al Kα anode operated with a photon energy of h = 1486.6 eV. A 400 µm × 400 µm spot size was used for sample analysis. A 50 eV pass energy was used for high-resolution scans. Charge neutralization and no sputtering was applied to acquire the data. Ten 10-µL aliquots were applied to a 99.99% Ta foil substrate of the PalCD-Co_3_O_4_ colloidal suspension, allowing for drying in between each application in preparation for those sample scans. Powder XRD was obtained using a Rigaku MiniFlex 600 diffractometer (Tokyo, Japan) operated at 600 W and 40 kV with a tube current of 15 mA, Cu Kα anode, and NaI scintillator counter. A goniometer scan rate of 1° 2θ/min was used. For the PalCD-Co_3_O_4_ composite, the same ten 10 µL application technique was used to apply the sample onto a zero-diffraction disk for analysis as used for the comparable XPS experiment (vide supra). Powders were mounted using electrically conductive double-sided tape, and blank scans were taken to ensure that no XRD peak signals emanated from it.

## Data Availability

The original contributions presented in this study are included in the article/Supplementary Materials. Further inquiries can be directed to the corresponding author.

## Supplementary Materials

The following supporting information can be downloaded at: https://www.mdpi.com/article/10.3390/molecules30020413/s1, Figure S1: Raman spectra showing integrated peak areas for (A) PalCDs, (B) RafCDs; and (C) SucCDs; Figure S2: SEM-EDX of PalCD-Co_3_O_4_ particulate; Figure S3: Randles–Sevçik analysis showing (A) CVs (raw data) of 100 µM DA in SU at 10 to 120 mV/s scan rates; and (B) linear plot; Figure S4: (A) stackplot of Raman spectra showing D and G bands of SucCD, SucCD-Co_3_O_4_; (B) LSVs of 10 to 80 µM in SU; and (C) exponential calibration plot of 10–100 µM DA in SU.
