# Supplementary material for: An Electrochemical Dopamine Assay with Cobalt Oxide Palatinose Carbon Dots
Source: Molecules. 2025 Jan 19;30(2):413. doi: 10.3390/molecules30020413 (PMC11767566; doi:10.3390/molecules30020413)
Supplement: Supplementary file 1 [file molecules-30-00413-s001.zip › molecules-3327609-supplementary.pdf]

Supplementary Materials

Electrochemically Assaying Dopamine with Cobalt Oxide Palatinose Carbon Dots

Ram Chandra Nepal,[1] Elif S. Seven,[2] Roger M. Leblanc,[2] and Charles C. Chusuei[1,*]

[1] Department of Chemistry, 440 Friendship Street, Middle Tennessee State University, Murfreesboro, TN 37132, USA; rcn2x@mtmail.mtsu.edu (R.C.N.)

[2] Department of Chemistry, University of Miami, 1301 Memorial Drive, Coral Gables, FL 33146, USA; ess133@miami.edu (E.S.S.); rml@miami.edu (R.M.L.)

* Correspondence: chusuei@mtsu.edu (C.C.C.); Tel.: 1-615-898-2079

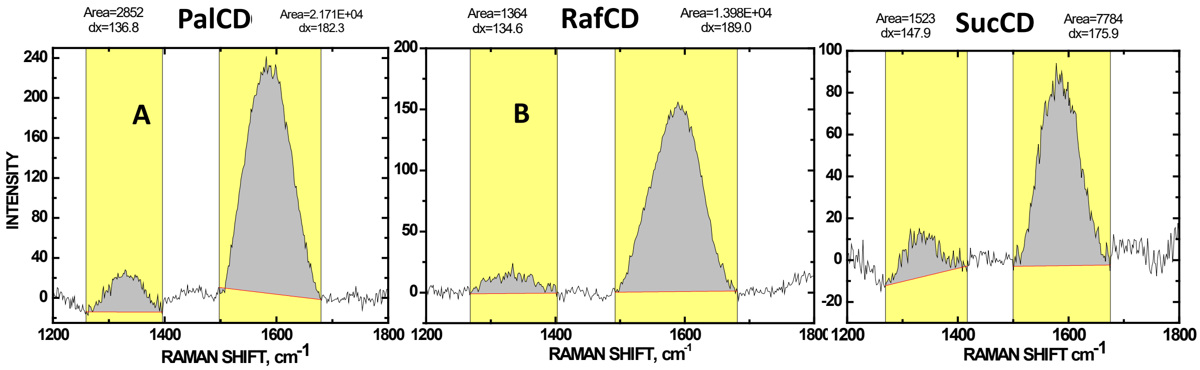

Figure S1: Raman peak areas for (A) PalCD, (B) RafCD and (C) SucCD.

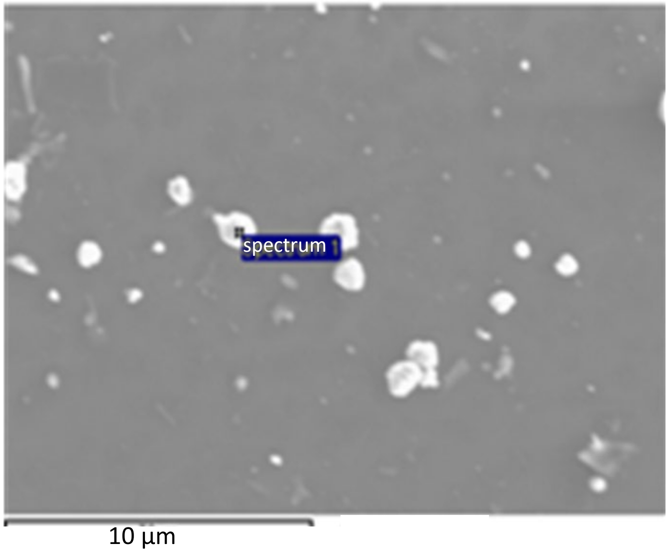

Spectrum 1 processing :

Peak possibly omitted : 2.617 keV

Processing option : All elements analyzed (Normalised)

Number of iterations = 5

Element	Weight%	Atomic%
C K	18.59	34.99
O K	32.81	46.36
Co K	48.60	18.65
Totals	100.00	

Figure S2: SEM-EDX of PalCD-Co_3O_4.

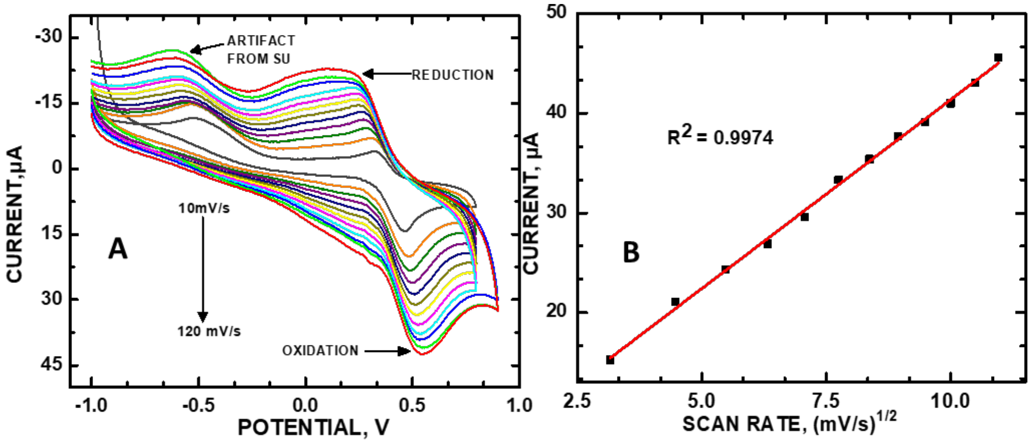

Figure S3: Randles-Sevçik analysis showing (A) CVs (raw data) of 100µM DA in SU at 10 to 120 mV/s scan rates; and (B) linear plot.

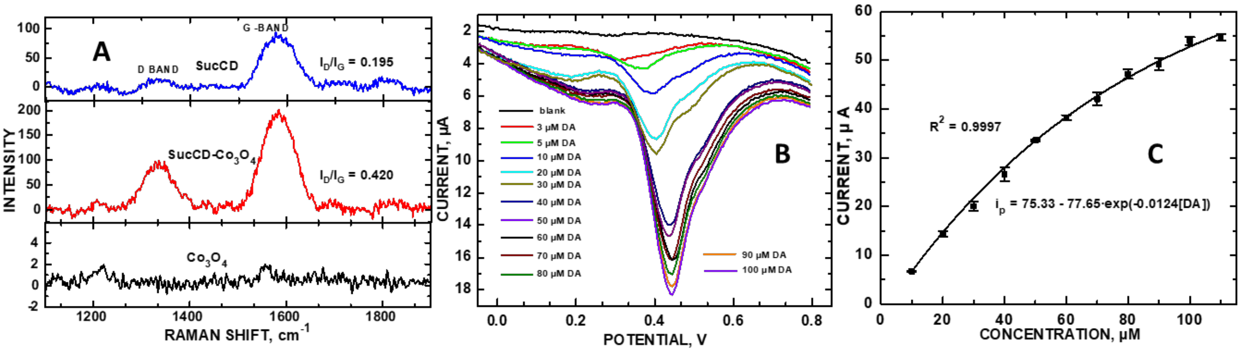

Figure S4: (A) stackplot of Raman spectra showing D and G bands of SucCD, SucCD-Co_3O_4, and Co_3O_4; (B) LSVs of 10 to 80 µM DA in SU; and (C) exponential calibration plot of 10-100 µM DA in SU.
